# Effect of Silver-Emitting Filler on Antimicrobial and Mechanical Properties of Soft Denture Lining Material

**DOI:** 10.3390/ma11020318

**Published:** 2018-02-22

**Authors:** Ewa Jabłońska-Stencel, Wojciech Pakieła, Anna Mertas, Elżbieta Bobela, Jacek Kasperski, Grzegorz Chladek

**Affiliations:** 1 Center of Dentistry and Implantology, ul. Karpińskiego 3, 41-500 Chorzów, Poland; eeewunia@gmail.com; 2 Faculty of Mechanical Engineering, Institute of Engineering Materials and Biomaterials, Silesian University of Technology, ul. Konarskiego 18a, 44-100 Gliwice, Poland; wojciech.pakiela@polsl.pl; 3 Chair and Department of Microbiology and Immunology, School of Medicine with the Division of Dentistry in Zabrze, Medical University of Silesia in Katowice, ul. Jordana 19, 41-808 Zabrze, Poland; amertas@sum.edu.pl (A.M.); ebobela@sum.edu.pl (E.B.); 4 Department of Prosthetic Dentistry, School of Medicine with the Division of Dentistry in Zabrze, Medical University of Silesia, pl. Akademicki 17, 41-902 Bytom, Poland; kroczek91@interia.pl

**Keywords:** antimicrobial properties, silver, soft lining, *Candida albicans*, denture, mechanical properties, sorption, solubility

## Abstract

Colonization of silicone-based soft lining materials by pathogenic yeast-type fungi is a common problem associated with the use of dentures. In this study, silver sodium hydrogen zirconium phosphate (SSHZP) was introduced into polydimethylsiloxane-based material as an antimicrobial filler at concentrations of 0.25, 0.5, 1, 2, 4, 6, 8, 10, 12, and 14% (*w*/*w*). The in vitro antimicrobial efficacy was investigated. *Candida albicans* was used as a characteristic representative of pathogenic oral microflora. *Staphylococcus aureus* and *Escherichia coli* were used as the typical Gram-positive and Gram-negative bacterial strains, respectively. The effect of filler addition on the Shore A hardness, tensile strength, tensile bond strength, sorption, and solubility was investigated. An increase in the filler concentration resulted in an increase in hardness, sorption, and solubility, and for the highest concentration, a decrease in bond strength. The favorable combination of antimicrobial efficacy with other properties was achieved at filler concentrations ranging from 2% to 10%. These composites exhibited mechanical properties similar to the material without the antimicrobial filler and enhanced in vitro antimicrobial efficiency.

## 1. Introduction

Soft denture lining materials are used in prosthetic dentistry to help in more even distribution of biting loads transmitted by dentures to soft tissues during chewing [1]. Additionally, soft lining materials possess the ability to absorb part of functional stress [2]. This may relieve the mucosa from a high, local mechanical stress, which supports prosthetic treatment [3]. Soft liners are usually recommended in patients with atrophied or sharp alveolar ridges, low tolerance of oral mucosa to pressure, atrophic mucosa, formation of recurrent sore spots under dentures on mucosa, poor retention of dentures, during implant healing [1,4,5], and for soft attachments for overdentures [6,7].

Soft linings can be categorized as tissue conditioners, intended for short-term use, and long-term soft linings, which may work in the oral cavity for more than four weeks, but they are usually used for many months or even several years [3,8]. Most of the currently available long-term soft linings are silicone rubbers, which are room temperature vulcanized (RTV) or heat temperature vulcanized (HTV). The former are more popular, typically made of polydimethylsiloxane-based composites reinforced with functionalized silica fillers. An important advantage of those dental materials is the possibility of relining using a standard laboratory procedure or with quick and easy chair-side technique (directly in the patient’s mouth). Materials based on other polymers are also commercially available, among which important are acrylates, where monomers like n-propyl and n-butyl methacrylate replace polymethyl methacrylate to lower glass transition temperature and decrease the required concentration of leachable plasticizers [8]. However, those materials’ properties are still less stable in comparison to silicones [3].

It is proven that the use of long-term soft linings improves speech as well as psychological comfort of patients, reduces feelings of pain, and increases retention and stability of dentures [9,10,11]. Moreover, in a comprehensive review, Palla et al. [12] conclude that, in particular, the use of long-term silicone soft liners considerably improves the mastication function compared to conventional hard poly(methyl methacrylate) (PMMA) denture bases. However, those materials also have some disadvantages. Silicone-based materials, due to their chemical composition, need adhesives to facilitate bonding to denture bases, and changes in bonding area may cause failures on the borders and loss of adhesion of the soft lining to the denture base. Another important problem is colonization by pathogenic yeast-like fungi within a short period. A clinical report has shown that *Candida* colonies may occur on the surface after the first three months, and during the next few months this becomes a serious problem, despite a good general hygiene of linings [13]. Numerous laboratory studies have been focused on different aspects of soft linings’ fungal colonization. Important factors contributing to colonization of soft linings by fungi include: using denture cleaners [14,15,16], presence of saliva and salivary proteins [17,18], a large number of thermal cycles [17,18], and low filler concentration [19]. The frequently investigated relationship between surface roughness and the adhesion of yeast-type fungi is still a cause of controversies, because some reports showed that smooth surfaces were less susceptible to colonization [20,21,22]. However, other works did not confirm this relationship [16,23]. Skupien et al. [24] reported that denture cleansers or irradiation with microwave energy decreased yeast counts on the surfaces, but complete elimination was not always reached. Additionally, in vitro studies proved that *Candida albicans* (*C. albicans*) colonize the surface and penetrate into silicone soft lining materials, which was first reported by Bulard et al. [25] and later confirmed by other studies [19,26]. These results suggested that the efficacy of typical cleaning agents might be limited, because the central section of denture soft linings can be a reservoir of *Candida* blastopores and hyphae forms [3]. This problem seems to be particularly important if we consider that a large number of denture wearers suffer from fungal infections in the oral cavity [27,28,29,30,31] and that the presence of soft liners may promote *Candida* colonization [20]. It is also known that the use of medications in older people may have negative outcomes [32] and frequent use of antifungal drugs may lead to drug resistance of pathogenic microorganisms [33]. Thus, to avoid complications related to the growth of microorganisms, denture base materials as well as soft denture lining materials with increased resistance against pathogenic fungi are required.

Different strategies have been used experimentally to increase the antifungal resistance of acrylic [34] and silicone [35,36,37] long-term soft denture lining materials with varying degrees of success. In this study, we report the use of silver sodium hydrogen zirconium phosphate (SSHZP) as a submicron filler for silicone matrix used as denture soft liners. SSHZP has been previously reported only as an antimicrobial additive into a PMMA denture base material, but it has not been tested in conjunction with silicone matrix, nor in the context of mechanical and physicochemical properties. SSHZP is a silver-emitting ceramic, but in contrast to silver nanoparticles, it is white. For that reason, it does not cause darkening of modified materials as a result of the plasmon effect of metal nanoparticles [37,38]. It is important because color is a vital functional property of the majority of dental materials. Therefore, the aim of this study was to evaluate the impact of SSHZP incorporation into silicone-based composites intended as denture soft linings on the materials’ antimicrobial effectiveness, hardness, tensile bond strength, tensile strength, sorption, and solubility. Our hypothesis was that composites filled with silver sodium hydrogen zirconium phosphate would show enhanced antimicrobial effectiveness and suitable properties for silicone denture soft lining materials.

## 2. Results

### 2.1. Scanning Electron Microscopy (SEM) Investigations

**Figure 1:**
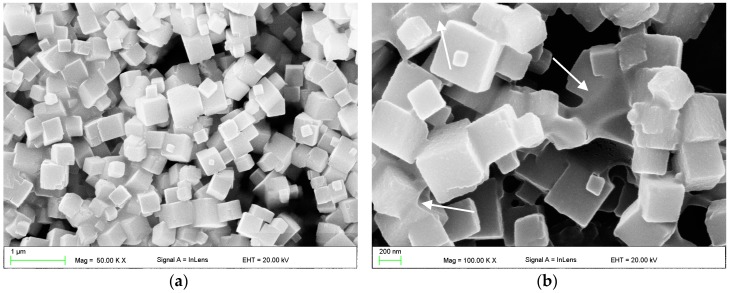
Scanning electron microscopy (SEM) images presenting the morphologies of (**a**) silver sodium hydrogen zirconium phosphate particles, and (**b**) their particles permanently connected to each other.

SEM images illustrating the morphology of a SSHZP powder are presented in Figure 1. The particular particles measured up to approximately 500 nm. However, particles permanently connected to each other were also observed (Figure 1b), which measured about 1 µm to 2 µm.

**Figure 2:**
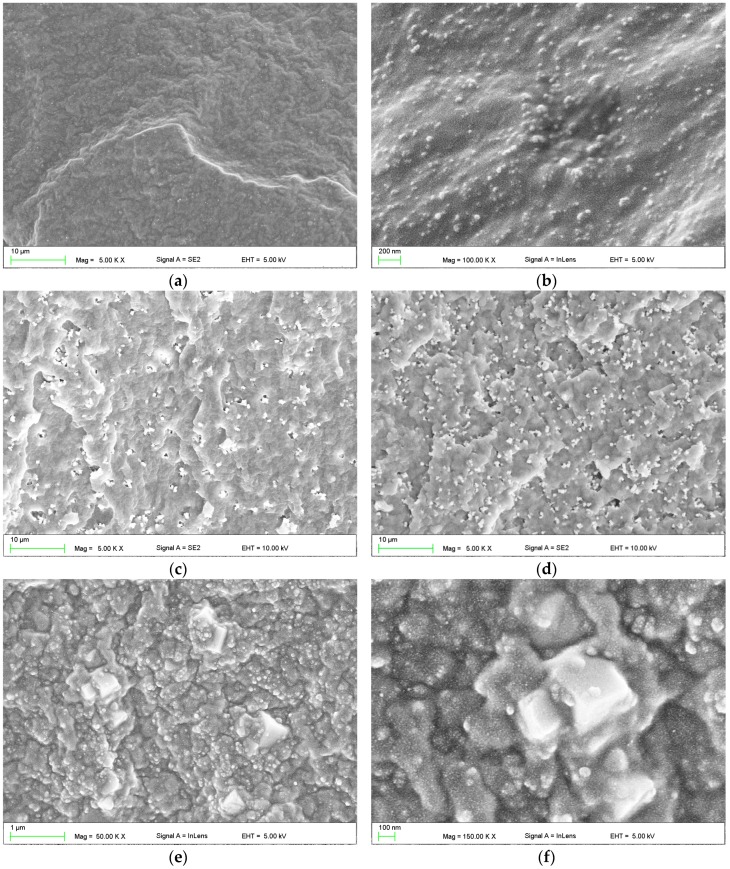
Representative SEM images presenting the morphologies of the crosslinked samples: (**a**,**b**) silicone composite reinforced with silica filler; (**c**,**d**) composites with additional antimicrobial filler concentrations of 6% and 14%, respectively; (**e**,**f**) aggregations or clusters of antimicrobial filler particles in the composite with the concentration of 12%.

SEM images illustrating the morphology of silicone material reinforced with 30% of silica filler (SF) and composites additionally compounded with SSHZP are presented in Figure 2. Starting material showed well-distributed silica filler particles or their aggregations, which measured from 20 nm to 100 nm (Figure 2b). When antimicrobial filler (AF) was compounded, it was in general well distributed in the matrix, and quality of the distribution was similar for all investigated materials (Figure 2c,d). Single particles were visible, however aggregations consisting of a few particles and measuring about 1 µm were frequently noted. Larger aggregations, measuring approximately 2 µm were usually observed starting at a concentration of 12%, and they were numerous at a concentration of 14%. Reinforcing silica filler particles and antimicrobial SSHZP particles were observed together only at higher magnification, and typical morphologies are presented in Figure 2e,f.

### 2.2. Microbiological Tests

The results of the antifungal and antibacterial tests are listed in Table 1. Positive and negative controls yielded expected results. Increasing the filler concentration had a significant effect (*p* = 0.0005) on the antimicrobial efficacy (AME) against *C. albicans*. For materials without SSHZP and for a SSHZP concentration of 0.25% to 0.5%, antifungal effects were not observed. For antimicrobial filler concentrations from 1% to 10%, the AME medians were from 48.4% to 68.5%, and they should be considered as similar if we take into account minimal and maximal AME values. Increasing the SSHZP concentration in the composite to 12% resulted in an additional increase in the AME median to 90.8%. For the highest examined concentration (14%), the maximum AME median was reached (98.8%), and the difference between minimal and maximal values was only 0.7%.

Increasing the filler concentration had also a significant effect (*p* < 0.0001) on the antimicrobial efficacy against *Staphylococcus aureaus* (*S. aureus*). For materials without SSHZP and for SSHZP concentrations from 0.25% to 1%, all antimicrobial efficacy medians were 0%. For antimicrobial filler concentrations from 2% to 6% the AME medians were from 58.7% to 71.6%, and due to the differences between minimal and maximal values, they should be considered as comparable. An additional increase in the SSHZP to concentrations from 8% to 10% resulted in a visible AME medians increase to 92.9% and 95.2%, respectively. Additionally, these results were more stable because of smaller differences between minimal and maximal AME values. Starting from antimicrobial filler concentrations of 12%, the AME medians were 100%.

Increasing the filler concentration had a significant effect (*p* < 0.0001) on the antimicrobial efficacy against *Escherichia coli* (*E. coli*). The antibacterial effects were noted starting from a SSHZP concentration of 2% and for all higher concentrations, AME medians were 100%.

### 2.3. Shore A Hardness

**Figure 3:**
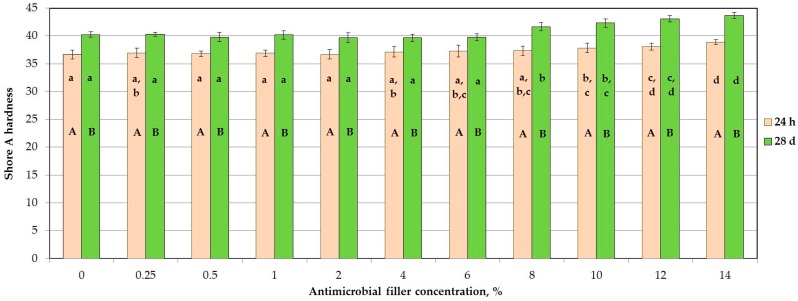
Mean hardness values in Shore A units and standard deviations after storing samples in distilled water; the different lowercase letters (a–d) for each material and uppercase letters (A–B) for each storing time show significantly different results at the *p* < 0.05 level.

The mean hardness values are presented in Figure 3. The SSHZP concentration had a significant influence on the hardness values of the composites (*p* < 0.0001). The hardness values increased with the antimicrobial filler concentration, however post hoc tests showed no statistically significant different mean values for starting material and the composites’ SSHZP concentrations from 0.25% to 6%. For concentrations starting from 8%, mean hardness values were statistically significantly (*p* < 0.05) greater than for materials without SSHZP, and they successively increased. However, the mean hardness values for the composite with antimicrobial filler concentration of 14% after 24 h and 28 days of storing were only 6% and 8% greater, respectively, than for the material without SSHZP. The hardness was observed to increase with storing time (*p* < 0.0001), and mean hardness values were from 3 to 5 Shore A units greater after four weeks in distilled water.

### 2.4. Tensile Strength

**Figure 4:**
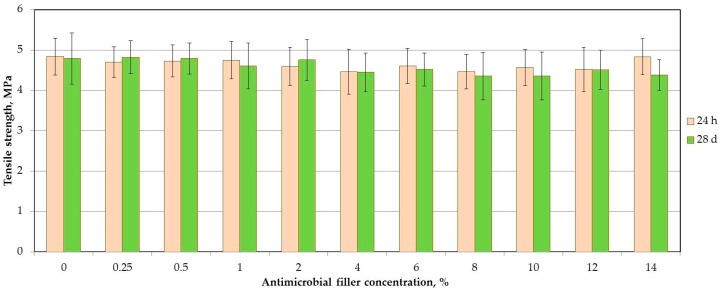
Mean values and standard deviations of tensile strength after storing samples in distilled water.

The mean tensile strength values are presented in Figure 4. The SSHZP concentration and storing time did not have a significant influence on the tensile strength of the materials (*p* = 0.0843 and *p* = 0.3319, respectively).

### 2.5. Tensile Bond Strength

**Figure 5:**
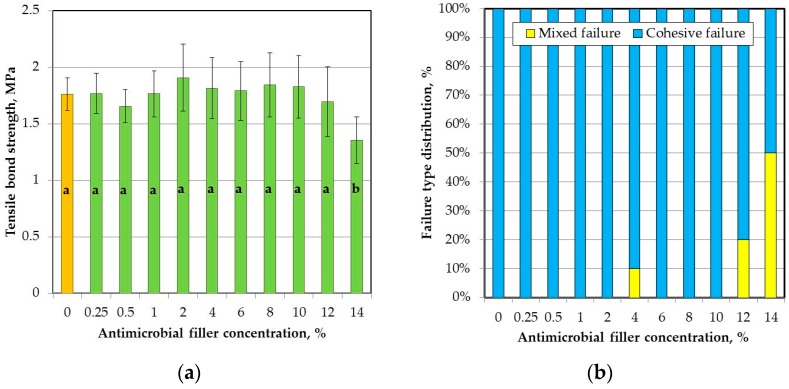
Results of bond strength test to the denture base material test: (**a**) mean and standard deviations of bond strength values (different lowercase letters (a–d) show significantly different results at the *p* < 0.05 level); (**b**) impact of the antimicrobial filler concentration on failure type.

**Figure 6:**
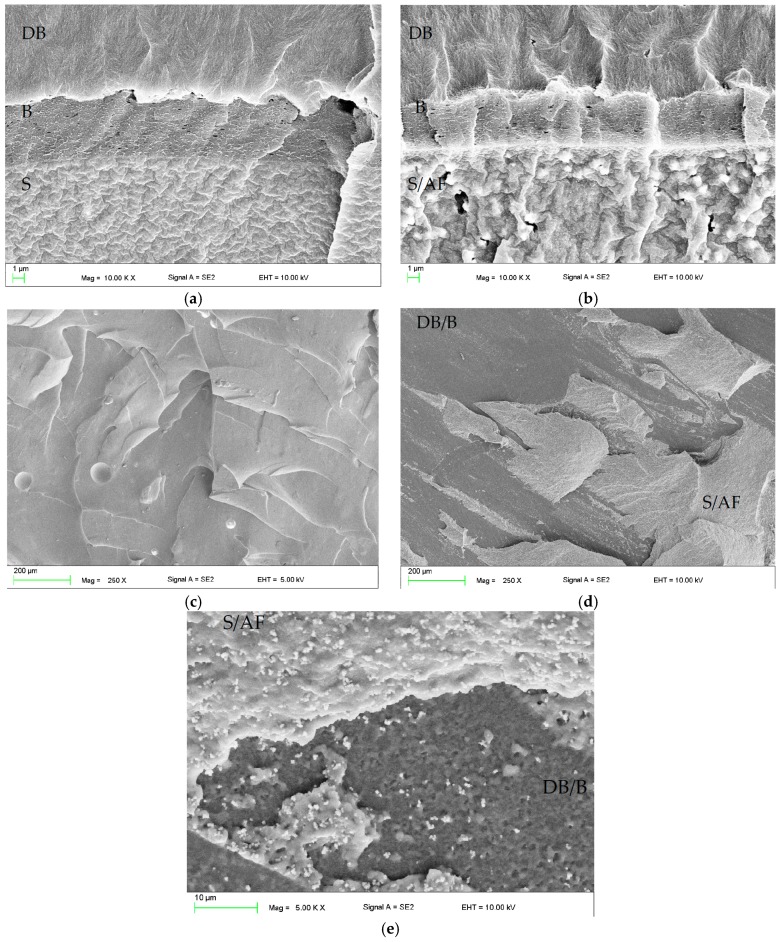
Representative SEM images presenting the morphologies of: (**a**,**b**) interface layer formed by bonding agent between poly(methyl methacrylate) (PMMA) resin and silica reinforced composite without antibacterial filler and with antimicrobial filler concentrations of 14% respectively; (**c**) morphology of cohesive failure; (**d**) morphology of mixed failure; (**e**) morphology of cohesive failure with visible SSHZP particles torn from the matrix and present on the surfaces of silicone and the bonding agent layer. DB—PMMA denture base material, B—layer formed by bonding agent, S—silicone material, S/AF—silicone material with antimicrobial filler, DB/B—areas of denture base material covered by bonding agent.

The mean values of tensile bond strength (T_B_) to the denture base resin are presented in Figure 5a. The SSHZP concentration had a significant influence on the T_B_ values (*p* = 0.0009), but the post hoc test showed a considerable decrease (*p* < 0.05) only for the highest concentration of 14%. The antimicrobial filler concentration also had a significant influence on the sample failure type (*p* < 0.0001). For the highest SSHZP concentration, 50% of failures were of mixed type, while for lower concentrations and unmodified material, adhesive failure was the only dominant failure type observed (Figure 5b). Thickness of layers created by bonding agent between silicone based composites and poly(methyl methacrylate) resin was similar for all materials (Figure 6a,b), and measured usually from 2 µm to 6 µm. SSHZP particles were not detected in this layer, however numerous particles or their aggregations were observed on the border between silicone composites and bonding agent (Figure 6b). The typical morphologies of cohesive and mixed (adhesive with cohesive) failures were presented in Figure 6c,d, respectively. At higher magnifications, a lot of SSHZP particles and their aggregations torn from the matrix were visible, and many of them were observed on the surface of the bonding agent layer, when “adhesive” areas of mixed failures were investigated (Figure 6e).

### 2.6. Sorption and Solubility

**Figure 7:**
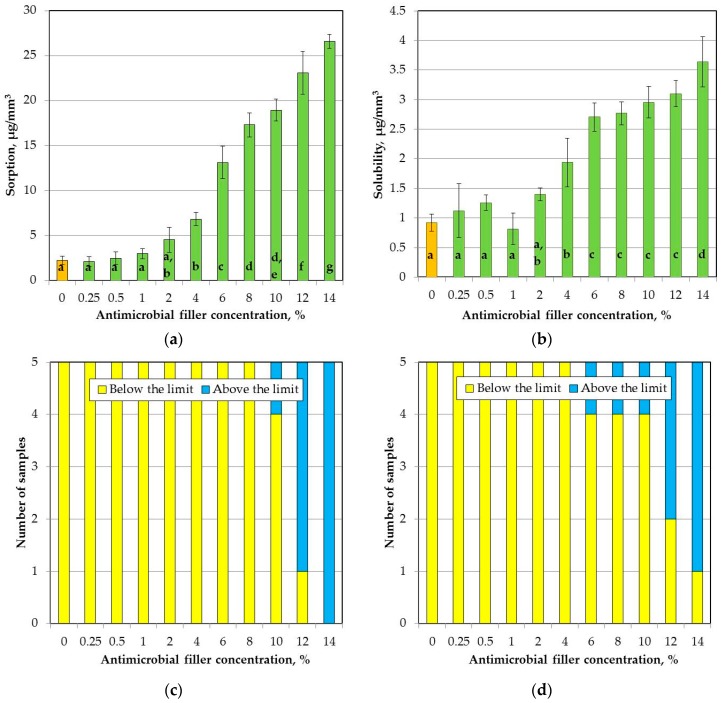
Mean and standard deviations of (**a**) sorption and (**b**) solubility (different lowercase letters (a–g) show significantly different results at the *p* < 0.05 level); numbers of samples for (**c**) sorption and (**d**) solubility values were below or above the limit required by the ISO standard.

The mean sorption values are presented in Figure 7a. The SSHZP concentration had a significant influence on sorption (*p* < 0.0001). The post hoc test showed a considerable (*p* < 0.05) and progressive increase in sorption starting from the antimicrobial filler concentration of 4%. For composites with concentrations of 12% and 14%, mean sorption values exceeded 20 µg/mm^3^, and additionally for four and five samples, respectively, showed values above this limit (Figure 7c).

The solubility increased significantly (*p* < 0.0001) with SSHZP concentration (Figure 7b). There were no significant differences (*p* > 0.05) in the mean solubility values obtained for materials up to a concentration of 2%. Next, solubility increased (*p* < 0.05). Between materials with concentrations from 6% to 12%, the differences were not statistically significant (*p* > 0.05), however, solubility slightly but progressively increased, and for the material with a SSHZP concentration of 12%, the mean value exceeded 3 µg/mm^3^. Additionally, for composites with concentrations of 12% and 14%, three and four samples, respectively, showed values above this limit. The solubility at the highest SSHZP content was statistically greater (*p* < 0.05) than for all other materials (Figure 7d).

## 3. Discussion

In the present study, experimental silicone-based composites were developed by introducing an additional filler: silver sodium hydrogen zirconium phosphate particles. The combination of mixing and ultrasonic homogenization allowed to obtain satisfactory dispersion of the antimicrobial filler, but aggregations were observed. However, in the compounded filler, numerous large structures, consisting of a few permanently connected particles, were also observed (Figure 1b). So, the question is, how many of these “aggregations” were in fact co-particles coming from the used SSHZP powder? The problems related with filler aggregation in the polymeric composites have been well-documented and reported for numerous laboratory-scale investigations [39], also for the PMMA denture base material modified with SSHZP [40].

Only a few works in the past years were focused on the possibilities of developing silicone soft lining materials with enhanced antifungal activity. Garner et al. [36] investigated antifungal coating composed of chlorhexidine nanoparticles on silicone rubber and showed inhibited metabolic activity of *C. albicans*, which is a very good prognostic before further investigations. This interesting method may allow in the future to renew the active layer when antimicrobial properties expire. Chladek et al. [37,41] introduced silver nanoparticles into a silicone soft lining and obtained enhanced antifungal activity, but higher concentrations of nanoparticles resulted in deterioration of various properties, including strong color changes. Pachava et al. [42] used tea tree oil at a concentration of 15% and reduced growth of *C. albicans*, however other properties of the obtained material are unknown. Perchyonok [43] modified a silicone soft lining material with copaiba oil and two different types of propolis. She obtained a zone of inhibition, but materials used in these investigations were uncured, so these findings need further confirmation in tests with crosslinked samples. Price et al. [35] chose another strategy and chemically modified the surface of an experimental silicone rubber. Incorporation of long-chain, hydrophilic or hydrophobic functional groups allowed to reduce the adherence of the *C. albicans* to the surfaces. In the current study, the experimental composites with different concentrations of SSHZP showed antimicrobial efficacy against *C. albicans* strain, which was used as a typical pathogenic microorganism related with using denture soft linings and denture stomatitis. The achieved activity against yeast was lower in comparison to results obtained with the same microbiological test for PMMA-based composites [40] containing SSHZP. However, the quality of filler dispersion in the current study was much better, which could affect the silver ion release into the environment. Antimicrobial properties of the SSHZP are activated under humid conditions by the mechanism of silver ion emission from an inorganic, insoluble carrier [44], so the number of particles contacted with the liquid, wet environment may be essential for the results. In the PMMA-based composite, numerous large aggregations were observed [40], which could lead to uncontrolled release of filler particles weakly bound to the matrix into microbial suspension and enhanced silver ion emission. Also, properties of the matrix, like sorption, solubility, and porosity, should be considered in the aspect of silver ions emission. This potential relation should be investigated in future. There is no point in directly comparing the obtained antimicrobial effectiveness with results for other antimicrobial agents, due to differences in microbiological tests protocols.

*E. coli* and *S. aureus* were used in this study as representative strains of Gram-positive and Gram-negative bacteria, respectively, and were not considered as typical bacteria associated with denture soft liners. However, it should be noted that some studies have concluded that bacteria, including *S. aureus* in combination with *C. albicans*, are probably responsible for denture stomatitis [45]. The presence of *S. aureus* on denture surfaces can also play a role in chronic inflammatory response in the oral mucosa, and in infections such as pneumonia [46,47,48]. Antibacterial activity against *S. aureus* was higher at the same SSHZP concentrations than for *E. coli*. This stays in accordance with reports where silver-containing materials [49,50,51,52,53] have shown a similar tendency. Lu et al. [50] suggested that these findings may be associated with the fact that cell walls of Gram-positive bacteria are thicker and more compact than cell walls of Gram-negative bacteria. The single-layered cell wall of a Gram-negative bacterium measures only 7–8 nm, whereas the cell wall of a Gram-positive bacterium consist of many peptidoglycan layers of approximately 40 nm to 80 nm [54].

Shore A hardness values after 24 h and 28 days of storing in distilled water were well below 50 Shore A units and 55 Shore A units, respectively, so they were within the specification limit for long-term soft lining materials [55]. Registered values and their changes were simultaneously just like those for commercially available silicone materials [56,57]. Increase in hardness with enhancing of antimicrobial filler concentration was at a low level (from 2 Shore A units to 3 Shore A units), and similar results were obtained by Han et al. [58], where different inorganic, not-silanized nanofillers were introduced into silicone elastomer. Slow hardening of composites during storing was registered, and these findings agree with those of Kim et al. [56], Mese et al. [57], and Iwaki et al. [59] for silicone soft linings. An increase in silicone hardness may occur because of continued crosslinking of investigated materials throughout the storing in distilled water at elevated temperature [60].

Tensile strength values were not significantly different after SSHZP introduction and were stable during four-week storing, which was especially favorable, because there was a threat that agglomerated particles will act as stress-concentrating centers in the silicone elastomer matrix and decrease the tensile strength [58]. These results were comparable to the values obtained for commercially available soft linings [61] and experimental maxillofacial silicone elastomers [62].

Tensile strength of silicone soft linings is not a standard property investigated for denture soft lining materials. In this study, it was investigated to show potential changes in composites during the first month in distilled water, especially in the context of deviations in tensile bond strength. Mutluay et al. [61] have shown a strong correlation between tensile strength and tensile bond strength. However, this relation is especially justified when cohesive failures are noted, because in these cases, the strength of the material itself is in fact lower than the strength of the interface layer formed by the bonding agent between PMMA resin and soft lining. This situation was noted in the present study up to a concentration of 10%. Starting from a concentration of 12%, mixed failures were observed, and for the concentration of 14%, additionally the mean tensile bond strength values were significantly lower. Simultaneously, tensile strength values were still stable, so it should be concluded that deterioration of the properties of the bonding area was related with the presence of particles and their aggregations near the borders of silicone-based composites and bonding agent (Figure 6c). Numerous particles and aggregations of SSHZP were also observed on the surface of the bonding agent of mixed failures (Figure 6e). These places were probably responsible for the reduction of contact area between composites and bonding agent or acted as stress-concentrating centers, which eventually resulted in decreased bond strength values. All obtained values of tensile bond strength were within the specification limit for long-term soft lining materials [55] and were similar to the results for long-term soft lining materials used nowadays [56,61].

Sorption and solubility of composites increased with SSHZP concentration. For composites with a concentration of 12% and 14%, more than one sample showed sorption and solubility values exceeding 20 µg/mm^3^ and 3 µg/mm^3^, respectively, so these materials did not meet the requirements of the ISO standard. Furthermore, up to antimicrobial filler concentration of 4%, sorption and solubility values were comparable to those registered for the available silicone soft lining materials [63,64,65]. The increase in the values of both properties should be considered as a disadvantage. The reasons of these unfavorable changes are unknown, but Kampmann et al. [44] reported that inorganic zirconium phosphate carrier for silver ions is insoluble, so the changes in sorption and solubility may be only partially related with the emission of ions (e.g., silver) from filler. However, the ion emission investigations were not carried out in the current study, so it should be done in the future. Potential changes in the matrix should also be tested. Furthermore, sorption was increased eleven-fold, while solubility was increased four-fold. This may suggest that one of the reasons of the noted changes could be insufficient coherence of the filler with the matrix, and migration of liquid inside the material associated with this. These assumptions, however, also require confirmation in the future.

The findings presented here must be enhanced with further in vitro investigations, and next confirmed with experiments conducted under in vivo conditions. As an important part of future research, the ions’ emission into environment and cytotoxic potential of composites should be examined. Admittedly, the toxicological data for the used filler [66] may suggest that composites should not present risks, despite some studies which showed that silver ions or nanoparticles may exhibit toxic effects in prosthetic devices [39]. In addition, studying the migration of silver ions from fillers in a polymer matrix can be combined with abrasion studies. When dentures are worn, more silver could be released from the polymer matrix through contact with the mucosa or while chewing, thereby temporarily improving antimicrobial activity. On the other hand, selective abrasion of the antimicrobial filler from the matrix may lead to an accelerated loss of the ability to release silver ions into the environment. Another remaining question is the influence of these suggested abrasion effects on the toxic potential of the materials. Mechanical cycling also leads to the surface degradation process and can change the surface texture of soft linings [67], which may affect the considered properties, too. Additionally, under clinical conditions numerous additional factors such as thermocycling, presence of saliva with salivary proteinases, and differentiated cleaning protocols of materials can influence the results, so the presented findings are only a promising starting point for further investigations.

## 4. Materials and Methods 

### 4.1. Materials Preparation 

Vinyl-terminated polydimethylsiloxane (PDMS), trimethylsiloxy-terminated methylhydrosiloxane–dimethylsiloxane copolymer (HPDMS) as crosslinker, platinum–divinyltetramethyldisiloxane complex as catalyst, and hexamethyldisilazane-treated amorphous silicon dioxide as a filler were purchased from Gelest (Morrisville, PA, USA). Silver sodium hydrogen zirconium phosphate (Milliken Chemical, Spartanburg, SC, USA) was used as an antimicrobial filler.

A mixture of 0.18% (*w*/*w*) of catalyst with vinyl-terminated polydimethylsiloxane was prepared by stirring with a magnetic stirrer at room temperature for 24 h. The antimicrobial filler and reinforcing filler were compounded in 50 mL Griffin form beakers at room temperature. Materials were prepared in 30 g portions. The concentration of silica filler in the mixture of PDMS and catalyst (without antimicrobial filler) was always 30% (*w*/*w*). The antimicrobial filler was compounded with concentrations of 0.25, 0.5, 1, 2, 4, 8, 10, 12, and 14% (*w*/*w*). Masses necessary to prepare materials were calculated according to the following equations:

m_AF_ = c_AF_ × m_c_ (1)

where m_AF_ was the silver sodium hydrogen zirconium phosphate mass, g; c_AF_ was the silver sodium hydrogen zirconium phosphate concentration, % (*w*/*w*); and m_c_ was the mass prepared composition of PDMS, catalyst, silica filler, and antimicrobial filler (always was 30 g).

m_SF_ = 30% × (m_c_ − m_AF_) (2)

where m_SF_ was the silica filler mass, g.

m_PDMS+C_ = 70% × (m_c_ − m_AF_) (3)

where m_PDMS+C_ was the mass of mixture PDMS with catalyst, g.

The AF was introduced as first. Fillers were introduced gradually in portions not greater than 0.5 g (they were smaller e.g., for low concentrations). After the addition of each portion, the composition was mechanically ground with a spatula on the wall of the beaker to apply shear forces, until a visually homogeneous consistency was achieved. After compounding, the whole mass of AF-prepared compositions was ultrasonically homogenized (Ultrasonic Homogenizer UP200St, Hielscher Ultrasonics GmbH, Teltow, Germany) in five series, each 90 ± 10 s, with intervals to cool in water at a temperature of 14 ± 4 °C. Next, the same protocol was used to compound SF. The obtained compositions were placed under the pressure of 80 mbar for 25 min in a vacuum stirrer (Twister evolution, Renfert, Hilzingen, Germany) to remove air bubbles.

In order to crosslink the composite samples, 4% (*w*/*w*) of HPDMS was added with an eyedropper and compositions were mixed in rapidly with a spatula. Mixed materials were quickly packed into stainless steel molds (excluding bond strength test) and loaded on a hydraulic press with the pressure of 0.3 MPa until the end of the setting time. During the crosslinking process, working time was approximately 1 minute and initial setting time was approximately 3 min. After the initial setting time, crosslinking of samples was continued in a prosthetic polymerizer (Palamat elite, Kulzer GmbH, Hanau, Germany) in distilled water under the pressure of 0.2 MPa at a temperature of 45 °C for 15 min.

### 4.2. SEM Investigations 

Samples for composite morphologies observations measured 40 mm × 10 mm × 6 mm and were crosslinked in a stainless steel mold, individually immersed in liquid nitrogen, broken, and sputtered with gold.

After tensile bond strength tests observations of failures, and cross-sections showing bonding zone between poly(methyl methacrylate) (PMMA) resin and silicone composites were carried out. Cross-sections were obtained by breaking samples in liquid nitrogen. All samples were sputtered with gold.

Investigations were realized using a Zeiss SUPRA 35 scanning electron microscope (Zeiss, Oberkochen, Germany) at accelerating voltages from 1 kV to 10 kV.

### 4.3. Microbiological Tests

The in vitro antimicrobial activities of the studied composites were examined according to the previously described method [40,68,69], with certain modifications. The following standard strains of microorganisms were used: the yeast-type fungus *Candida albicans* ATCC 10231 (*C. albicans*), Gram-negative *Escherichia coli* ATCC 25922 (*E. coli*), and Gram-positive *Staphylococcus aureus* ATCC 25923 (*S. aureus*). Sterilized square specimens that measured 10 mm × 10 mm and were 2 mm *thick of the* studied composites were introduced individually in 2 mL of fungal or bacterial suspensions in tryptone water, which contained approximately 1.5 × 10^5^ CFU/mL (CFU, colony forming units) of *C. albicans, E. coli*, or *S. aureus*. Suspensions of bacteria or fungi in tryptone water were tested as a positive control. As a negative control, pure tryptone water was tested. All of the samples with microorganism suspensions were incubated in a shaking incubator for 17 h at 35 °C for *C. albicans* and at 37 °C for *S. aureus* and *E. coli*. After incubation, 20 μL of suspension was seeded onto Columbia agar with 5% sheep blood plates for *S. aureus*, MacConkey agar plates for *E. coli*, and Sabouraud agar plates for *C. albicans*. The Columbia agar, Sabouraud agar, and MacConkey agar were purchased from bioMerieux (Marcy l’Etoille, France). The cultured plates were incubated at 35 °C for 48 h (yeast) or 37 °C for 24 h (bacteria). Then, the number of fungal or bacterial colonies were counted [70]. The materials’ antimicrobial efficacy was calculated according to the following equation:

(4) $$\mathrm{AME}=\frac{V_{c}-V_{t}}{V_{c}}$$

where V_c_ was the number of viable microorganisms colonies of the positive control (BLANK), and V_t_ was the number of viable microorganisms colonies of the test specimen.

### 4.4. Hardness Tests

The hardness after 5 s of loading was measured using a method presented in the ISO standard [55]. A Shore A digital durometer (Bareiss HPE II-A, Bariess, Oberdischingen, Germany) was used to measure the hardness. Three samples measuring 40 mm in diameter and 6 mm in thickness for every material were crosslinked in a stainless steel mold. The hardness was measured after 24 h and 28 days of storing in distilled water at 37 ± 1 °C at five points of every sample after each time.

### 4.5. Tensile Strength Tests

For tensile strength tests, dumbbell-shaped samples of type 4 specified by the ISO standard [71] for rubbers were cut off from 2 mm thick cured plates [72] with a manual cutting press (ZCP 020, Zwick, Ulm, Germany). From one plate two samples were made, each for one storing time in distilled water at 37 ± 1°C (24 h or 28 days), and twenty samples were manufactured for every composite material. After storing, samples were removed from water, measured, and tensile testing was performed using a universal testing machine (Zwick Z020, Zwick GmbH & Com, Ulm, Germany). A cross-head speed was 10 mm/min [61]. Ultimate tensile strength was calculated according to the following equation:

(5) $$T_{S}=\frac{F}{A}$$

where: T_S_ is ultimate tensile strength, MPa; F is force at rupture, N; and A is the initial cross-sectional area of specimen, mm^2^.

### 4.6. Tensile Bond Strength Tests

The tensile bond strength (T_SB_) to the denture base resin was examined with a method presented in the ISO standard [55] with certain specifications regarding the preparation of samples [41]. Samples of PMMA resin (Vertex Rapid Simplified, Vertex-Dental B.V., Zeist, The Netherlands) measuring 25 ± 2 mm on the side and 3.2 ± 0.2 mm in thickness were polymerized in accordance with the manufacturer’s instructions with a standard flasking technique. The surfaces of pieces were wet-ground with P500-grit abrasive paper and conditioned in distilled water at 37 ± 1 °C for 28 days. After conditioning, the samples were removed from water in pairs. The surfaces of the PMMA samples were dried and the bonding agent (Sofreliner Tough M Bonding, Tokuyama, Tokyo, Japan) was applied with a brush. A polyethylene ring (a height of 3 ± 0.1 mm and the internal diameter of 11 mm) was hurriedly placed in the center of the first PMMA sample, the composite material was rapidly manually mixed and injected into the ring, then a second PMMA sample was placed over the composite and compressed. Ten samples were prepared from each composite. When the crosslinking procedure was finished, the reducers mounted in the jaws of the universal testing machine were used to fix the handles (M4 screws) to specimens by the cold-curing PMMA resin (Vertex Castapress, Vertex-Dental B.V., Zeist, The Netherlands). Next, the samples were placed in distilled water for 24 ± 1 h at 37 ± 1 °C. After removing from water, each sample was mounted in reducers again, placed in the jaws of the testing machine, and tensile test was performed at a cross-head speed of 10 mm/min. Tensile bond strength was calculated with the use of the following equation:

(6) $$T_{\mathrm{SB}}=\frac{F_{m}}{A}$$

where: T_SB_ is tensile bond strength, MPa; F_m_ is maximal force, N; and A is cross-sectional area of the internal section of the polyethylene ring, mm^2^.

### 4.7. Sorption and Solubility

Sorption and solubility were examined using a method presented in the ISO standard [55]. Five test samples of each material, measuring 0.5 mm in thickness and 50 mm in diameter, were crosslinked in stainless steel molds. The samples were weighed (Analytic Scale 110/C/2, Radwag, Poland) with an accuracy of 0.1 mg and dried inside desiccators with freshly dried silica gel in a dryer at 37 ± 1 °C. When the daily changes in mass were no higher than 0.2 mg (mass values were recorded as m_1_), the thickness and diameter of samples were measured with a digital caliper with an accuracy of 0.01 mm and samples were placed in distilled water for 7 days at 37 ± 1 °C. After storing, the samples were removed from water singly, dried from visible moisture with filter paper, rapidly air-dried, and then weighed. The recorded mass was denoted as m_2_. The samples were again dried as described above and stable mass was denoted as m_3_. Sorption and solubility were calculated using the following equations:

(7) $$w_{\mathrm{sp}}=\frac{m_{2}-m_{3}}{V}$$

(8) $$w_{\mathrm{sl}}=\frac{m_{1}-m_{3}}{V}$$

where w_sp_ is sorption; w_sl_ is solubility; m_1_ is the initial mass of dried sample, µg; m_2_ is the mass after storing, µg; m_3_ is the mass after the second drying, µg; and V is the volume of the sample, mm^3^.

### 4.8. Statistical Analyzes

Statistical analysis of the results was performed with the use of the Statistica 13.1 software (TIBCO Software Inc., Palo Alto, CA, USA). The distributions of the residuals were tested with Shapiro-Wilk test, and the equality of variances was tested with Bartlett test. When the distribution of the residuals was normal and the variances were equal, the one-way or two-way ANOVA with Tukey HSD post hoc tests were used (α = 0.05), otherwise the non-parametric Kruskal-Wallis test (α = 0.05) was used. The impact of the AF concentration on the type of failure observed was defined by the Pearson’s chi-square (χ^2^) test for categorical data (α = 0.05). The results of microbiological tests were statistically evaluated using non-parametric Kruskal-Wallis test (α = 0.05).

## 5. Conclusions

Within the limits of this study, it can be concluded that the PDMS-based material was successfully modified with antimicrobial filler, and the obtained composites showed a high initial antimicrobial efficacy for the tested fungi and bacteria. The favorable combination of antimicrobial efficacy with other properties was achieved for filler concentrations ranging from 6% to 10%. Those materials, considered as denture long-term soft linings, exhibited mechanical properties similar to reference material as well as sorption and solubility at acceptable level. Further long-term studies of the migration of silver ions from composites to the environment, durability of the achieved antimicrobial efficacy, and cytotoxic tests need to be performed. Future results should be coupled with the presented research.

## Figures and Tables

**Table 1:** Antimicrobial efficacy (AME) against *Candida albicans* ATCC 10231, *Escherichia coli* ATCC 25922 and *Staphylococcus aureus* ATCC 25923 after 17 h of incubation with samples of the experimental materials.

Filler Concentration, %	Antimicrobial Efficacy, %								
	*Candida albicans* ATCC 10231			*Staphylococcus aureus* ATCC 25923			*Escherichia coli* ATCC 25922		
	Med	Max	Min	Med	Max	Min	Med	Max	Min
0	−7.5	3.7	−18.5	0.0	1.8	0.0	0.0	0.0	0.0
0.25	4.8	7.2	−15.5	0.0	0.0	0.0	0.0	0.0	0.0
0.5	−6.3	0.6	−16.9	0.0	0.0	0.0	0.0	0.0	0.0
1	48.4	51.7	29.2	0.0	24.6	0.0	0.0	0.0	0.0
2	56.6	70.9	30.7	58.7	100.0	23.5	>99.9	>99.9	>99.9
4	50.6	53.9	45.5	71.6	90.4	39.5	100.0	100.0	100.0
6	63.1	71.4	56.0	65.1	90.4	56.4	100.0	100.0	100.0
8	45.6	63.1	34.9	92.9	96.2	81.0	100.0	100.0	100.0
10	68.5	82.2	47.4	95.2	97.0	87.9	100.0	100.0	100.0
12	90.8	99.1	65.7	100.0	100.0	100.0	100.0	100.0	100.0
14	98.8	99.3	98.6	100.0	100.0	100.0	100.0	100.0	100.0

Med—median, Min—minimal value, Max—maximal value.
